# Phase II study of mFOLFOX6 in advanced gastric cancer patients with severe peritoneal metastases (WJOG10517G), including an individual patient data comparison with JCOG1108/WJOG7312G

**DOI:** 10.1007/s10120-026-01790-3

**Published:** 2026-08-27

**Authors:** Toshiki Masuishi, Hiroki Hara, Takayuki Ando, Takeshi Kawakami, Yoshiyuki Yamamoto, Naotoshi Sugimoto, Kazuhiro Shiraishi, Taito Esaki, Yuji Negoro, Takao Tsuzuki, Hiroaki Sawai, Masato Nakamura, Takashi Inagaki, Yudai Shinohara, Kentaro Kawakami, Seiichiro Mitani, Kenji Tsuchihashi, Chihiro Kondoh, Hiroo Katsuya, Osamu Maeda, Yuriko Fujita, Kenichi Yoshimura, Masanori Terashima, Takako Eguchi Nakajima, Kei Muro

**Affiliations:** 1 Department of Clinical Oncology, Aichi Cancer Center Hospital, 1-1 Kanokoden, Chikusa-ku, Nagoya, Aichi 464-8681 Japan; 2 Third Department of Internal Medicine, University of Toyama, Toyama, Japan; 3 Department of Gastroenterology, Saitama Cancer Center, Saitama, Japan; 4 Division of Gastrointestinal Oncology, Shizuoka Cancer Center, Shizuoka, Japan; 5 Department of Gastroenterology, Faculty of Medicine, University of Tsukuba, Tsukuba, Japan; 6 Department of Genetic Oncology, Osaka International Cancer Institute, Osaka, Japan; 7 Department of Medical Oncology, NHO Nagoya Medical Center, Nagoya, Japan; 8 Department of Gastrointestinal and Medical Oncology, National Hospital Organization Kyushu Cancer Center, Fukuoka, Japan; 9 Department of Oncological Medicine, Kochi Health Sciences Center, Kochi, Japan; 10 Department of Internal Medicine, Japanese Red Cross Society Himeji Hospital, Himeji, Japan; 11 Department of Gastroenterology, Takatsuki General Hospital, Takatsuki, Japan; 12 Aizawa Comprehensive Cancer Center, Aizawa Hospital, Matsumoto, Japan; 13 Department of Medical Oncology and Hematology, Faculty of Medicine, Oita University, Oita, Japan; 14 Department of Hematology and Oncology, Japan Community Healthcare Organization Kyushu Hospital, Kitakyushu, Japan; 15 Department of Medical Oncology, Keiyukai Sapporo Hospital, Sapporo, Japan; 16 Department of Medical Oncology, Faculty of Medicine, Kindai University, Sakai, Japan; 17 Department of Hematology, Oncology and Cardiovascular Medicine, Kyushu University Hospital, Kyushu, Japan; 18 Department of Medical Oncology, Toranomon Hospital, Tokyo, Japan; 19 Division of Hematology, Respiratory Medicine and Oncology, Department of Internal Medicine, Faculty of Medicine, Saga University, Saga, Japan; 20 Department of Clinical Oncology and Chemotherapy, Nagoya University Hospital, Nagoya, Japan; 21 Department of Gastroenterology, Yokohama Municipal Citizen’s Hospital, Yokohama, Japan; 22 Division of Gastric Surgery, Shizuoka Cancer Center, Shizuoka, Japan; 23 Innovative Clinical Research Center (iCREK), Nagoya City University, Nagoya, Japan; 24 Department of Early Clinical Development, Kyoto University Graduate School of Medicine, Kyoto, Japan

**Keywords:** Gastric cancer, Severe peritoneal metastases, Massive ascites, Inadequate oral intake, FOLFOX

## Abstract

**Background:**

Advanced gastric cancer patients with severe peritoneal metastases, defined as massive ascites throughout the abdominal cavity and/or inadequate oral intake requiring intravenous infusion support, have been excluded from pivotal clinical trials.

**Methods:**

This phase II trial evaluated mFOLFOX6 in HER2-negative, chemotherapy-naive advanced gastric cancer patients with severe peritoneal metastases. The primary endpoint was overall survival (OS); secondary endpoints included ascites response rate among patients with massive ascites and oral intake improvement rate. A sample size of 50 was planned to detect a median OS of 9 months, with a threshold of 6 months (one-sided α = 0.05; β = 0.2). A prespecified individual patient data comparative analysis was performed using data from patients treated with 5-fluorouracil/l-leucovorin (FL) or FL plus paclitaxel (FLTAX) in JCOG1108/WJOG7312G.

**Results:**

Among 51 patients, Eastern Cooperative Oncology Group performance status was 0/1/2 in 3/40/8 patients. Median OS and progression-free survival were 7.4 (lower limit of one-sided 95% confidence interval [CI], 6.5) and 3.8 months, respectively. Among 25 patients with massive ascites, the ascites response rate was 36%. Of 33 patients with inadequate oral intake, 46% achieved improved oral intake. The most frequent grade 3–4 adverse event was neutropenia (51%), and febrile neutropenia occurred in one patient (2%). No treatment-related deaths occurred. In propensity score-weighted analysis, OS hazard ratios with mFOLFOX6 versus FL and FLTAX were 0.76 (95% CI 0.56–1.03) and 0.87 (95% CI 0.64–1.18), respectively.

**Conclusions:**

mFOLFOX6 was tolerable and may be a treatment option for advanced gastric cancer patients with severe peritoneal metastases.

**Clinical trial registration:**

jRCTs041180007.

**Supplementary Information:**

The online version contains supplementary material available at https://doi.org/10.1007/s10120-026-01790-3.

## Introduction

Currently, fluoropyrimidine and oxaliplatin, with or without anti-PD-1 antibodies, are the recommended first-line treatment for patients with human epidermal growth factor receptor 2 (HER2)-negative advanced gastric cancer (AGC) [1–3]. For HER2-positive [4] and CLDN18.2-positive [5, 6] AGC, trastuzumab and zolbetuximab, respectively, are added to fluoropyrimidine and platinum-based regimens.

Peritoneal metastasis is the most frequent metastatic pattern in gastric cancer, often resulting in ascites, bowel obstruction, or paralytic ileus. Approximately 10% of previously untreated AGC patients present with severe peritoneal metastases (SPM), defined as massive ascites and/or inadequate oral intake [7]. These patients were excluded from pivotal trials such as CheckMate 649 [1] and ATTRACTION-4 [2], which compared fluoropyrimidine, oxaliplatin, and nivolumab versus fluoropyrimidine and oxaliplatin, due to their poor prognosis and tumor-related complications. As a result, standard treatment development for this subgroup has progressed separately. The JCOG1108/WJOG7312G randomized phase II/III trial comparing 5-fluorouracil/l-leucovorin (FL) with FL plus paclitaxel (FLTAX) was the first to demonstrate that AGC patients with SPM could safely receive chemotherapy [8]. However, median overall survival (OS) remained limited at around 6–7 months, and their prognosis remains extremely poor. Retrospective studies of mFOLFOX6 reported promising efficacy, with median OS ranging from 8.8 to 13.2 months, and showed benefit in improving ascites and oral intake in patients with SPM [7, 9]. Still, these studies involved small sample sizes and included patients who received mFOLFOX6 with initial dose modifications, leaving the efficacy and safety of mFOLFOX6 in this setting uncertain.

Therefore, we conducted a phase II study of mFOLFOX6, including a safety lead-in part in AGC patients with SPM [10]. To address the limitations of this single-arm phase II trial, we also performed a prespecified individual patient data comparative analysis using data from the JCOG1108/WJOG7312G study (FLTAX and FL arms).

## Materials and methods

### Study design and treatment schedule

This multicenter, open-label, single-arm phase II trial evaluated the efficacy and safety of mFOLFOX6 in AGC patients with SPM. The study included a safety lead-in part to assess safety during the first cycle of mFOLFOX6 in the initial six patients. Dose-limiting toxicities (DLTs) in safety lead-in part were defined as febrile neutropenia; grade 4 neutropenia lasting >7 days; grade 4 thrombocytopenia or platelet transfusion at physician discretion; grade ≥3 nonhematologic toxicities; or adverse events requiring treatment delay of 15 days or more from the planned start date. Following confirmation of safety in these six patients, full enrollment resumed.

The mFOLFOX6 regimen comprised oxaliplatin 85 mg/m^2^ and l-leucovorin 200 mg/m^2^ administered concurrently as 2 h intravenous infusion, followed by a 400 mg/m^2^ bolus of 5-FU and then a 2,400 mg/m^2^ continuous infusion of 5-FU over 46 h, repeated every 2 weeks. Treatment continued until disease progression, unacceptable toxicity, death, or patient withdrawal of consent.

The study was conducted in accordance with the Declaration of Helsinki and the Clinical Trials Act in Japan. All patients provided written informed consent before participation. The study protocol was approved by the Certified Review Board (Aichi Cancer Center Hospital, CRB4200002) and registered with the Japan Registry of Clinical Trials (Trial ID: jRCTs041180007).

### Eligibility criteria

The main eligibility criteria were as follows: (1) age between 20 and 75 years; (2) histologically confirmed adenocarcinoma; (3) HER2-negative status if tested, or untested/unanalyzable; (4) unresectable or recurrent gastric cancer with peritoneal metastasis; (5) either i) Eastern Cooperative Oncology Group (ECOG) Performance Status (PS) of 0–1 with massive ascites and/or inadequate oral intake, or ii) ECOG PS of 2 with massive ascites or inadequate oral intake; (6) no prior chemotherapy for AGC; and (7) adequate bone marrow, hepatic, and renal function. Peritoneal metastasis was defined by one or more of the following: induration detected by digital rectal examination; gastrointestinal stenosis or obstruction identified by gastrointestinal series; peritoneal nodules, hydronephrosis, extrahepatic bile duct dilation, or ascites not attributable to other causes, as detected on computed tomography (CT); and cytologically or pathologically confirmed malignant ascites or peritoneal involvement. Massive ascites was defined as continuous ascites extending on CT from the pelvic cavity to the upper abdomen, including the perihepatic and/or perisplenic regions. The assessment was performed by investigators at each participating institution without central radiological review. Inadequate oral intake was defined as a clinical requirement, as determined by the treating physician, for intravenous infusion support to maintain adequate hydration and nutrition because oral intake alone was insufficient. No minimum duration or volume of intravenous infusion was prespecified. This criterion was intended to identify patients expected to require ongoing intravenous support unless the underlying cancer-related condition causing inadequate oral intake improved. These definitions were consistent with those used in the JCOG1108/WJOG7312G study [8].

### Outcome measures

The primary endpoint was OS. Secondary endpoints included progression-free survival (PFS), response rate (RR), disease control rate (DCR), time to treatment failure (TTF), improvement in oral intake among patients with inadequate intake at enrollment, ascites drainage-free survival, ascites response and control rates among patients with ascites at enrollment, dose intensity, and incidence of adverse events. Efficacy was evaluated using the Response Evaluation Criteria in Solid Tumors version 1.1, based on CT scans performed every 8 weeks. OS was defined as the time from enrollment to death from any cause. PFS was defined as the time from enrollment to either disease progression or death from any cause. RR was the proportion of patients achieving a complete or partial response. DCR was defined as the proportion of patients achieving complete response, partial response, or stable disease. TTF was measured from enrollment to treatment discontinuation or death from any cause. Improvement in oral intake was defined as maintaining adequate oral intake for ≥7 days without the need for daily intravenous infusion. Ascites drainage-free survival was defined as the time from enrollment to either the first ascites drainage or death from any cause. Ascites levels were assessed by CT and categorized as follows: massive, continuous ascites extending from the pelvic cavity to the upper abdomen; moderate, ascites that was neither massive nor mild; mild, ascites confined to the pelvic cavity or upper abdomen; and *none*, no detectable ascites. Ascites response was classified according to changes in these prespecified levels: ascites complete response (ascites CR) was defined as disappearance of ascites; ascites partial response (ascites PR) as a decrease by at least one ascites level; ascites stable disease (ascites SD) as no change in ascites level; and ascites progressive disease (ascites PD) as an increase by at least one ascites level. An ascites assessment was classified as not evaluable (ascites NE) if an ascites-directed procedure was performed after initiation of protocol treatment or if CT assessment was not performed. The best ascites response was determined according to the prespecified order of ascites CR > ascites PR > ascites SD > ascites PD > ascites NE, from most to least favorable. Ascites response was assessed by investigators at each participating institution without central radiological review. Toxicity was assessed according to the National Cancer Institute Common Toxicity Criteria version 4.0.

A prespecified individual patient data comparative analysis was conducted between patients enrolled in this study and those from the JCOG1108/WJOG7312G study (Clinical trial information: UMIN000010949) who received either FLTAX or FL. In the JCOG1108/WJOG7312G study, patients with an ECOG PS of 2 along with both massive ascites and inadequate oral intake were determined to be ineligible after patients were enrolled because early deaths occurred in these patients. Therefore, for the present analysis, patients meeting those ineligibility criteria were excluded from the FL and FLTAX cohorts, ensuring that eligibility and exclusion criteria were completely consistent across both studies. For the comparative safety analysis, adverse events were summarized descriptively according to the worst CTCAE grade during the observation period among patients who met the WJOG10517G eligibility criteria and initiated protocol treatment.

### Statistical analysis

Because OS of 6 months was previously reported in AGC patients with SPM treated with FL [11], which was considered the standard treatment at the time this study was planned, we set a threshold OS of 6 months. Based on this, we calculated a target sample size of 50 patients, including six from the safety lead-in part. An OS of 9 months was considered promising, while 6 months was deemed unacceptable (one-sided α = 0.05; β = 0.2).

OS, PFS, TTF, and ascites drainage-free survival were estimated using the Kaplan–Meier method. Confidence intervals (CIs) for median values were calculated using the Brookmeyer–Crowley method. RR, DCR, rate of improvement in oral intake, ascites response and control rates, and incidence of adverse events were calculated using the binomial distribution, with CIs accordingly derived. For the individual patient data comparative analysis, unadjusted hazard ratios (HRs) for OS and PFS were estimated using Cox proportional hazards models. Propensity scores were estimated using a binary logistic regression model, with study cohort (WJOG10517G vs. JCOG1108/WJOG7312G) as the dependent variable, to adjust for differences in baseline characteristics between the two studies. The FL and FLTAX groups were pooled for propensity score estimation because patients were randomly assigned to these treatments within JCOG1108/WJOG7312G. The model included sex; ECOG performance status (0–1 vs. 2); number of factors contributing to SPM (massive ascites and/or inadequate oral intake; 1 vs. 2); disease status (unresectable vs. recurrent); histological type (diffuse vs. intestinal); white blood cell count (≥ 8,600/μL vs. <8,600/μL); alkaline phosphatase level (≥ 322 U/L vs. <322 U/L); and modified Glasgow Prognostic Score (mGPS; 0–1 vs. 2) as covariates. Patients with missing values for any of these covariates were excluded from the propensity score-weighted analysis. Inverse probability of treatment weighting (IPTW) using unstabilized weights was applied based on the estimated propensity scores to estimate the average treatment effect in the combined study population. After weighting, FL, FLTAX, and mFOLFOX6 were analyzed as three separate treatment groups. Adjusted HRs for OS and PFS, with 95% CIs and *p* values, were estimated using weighted Cox proportional hazards models and Wald tests. Model-based variances derived from the partial likelihood were used for variance estimation.

All statistical analyses were conducted using SAS version 9.4 (SAS Institute, Cary, N.C., USA).

## Results

### Patient characteristics

**Fig. 1:**
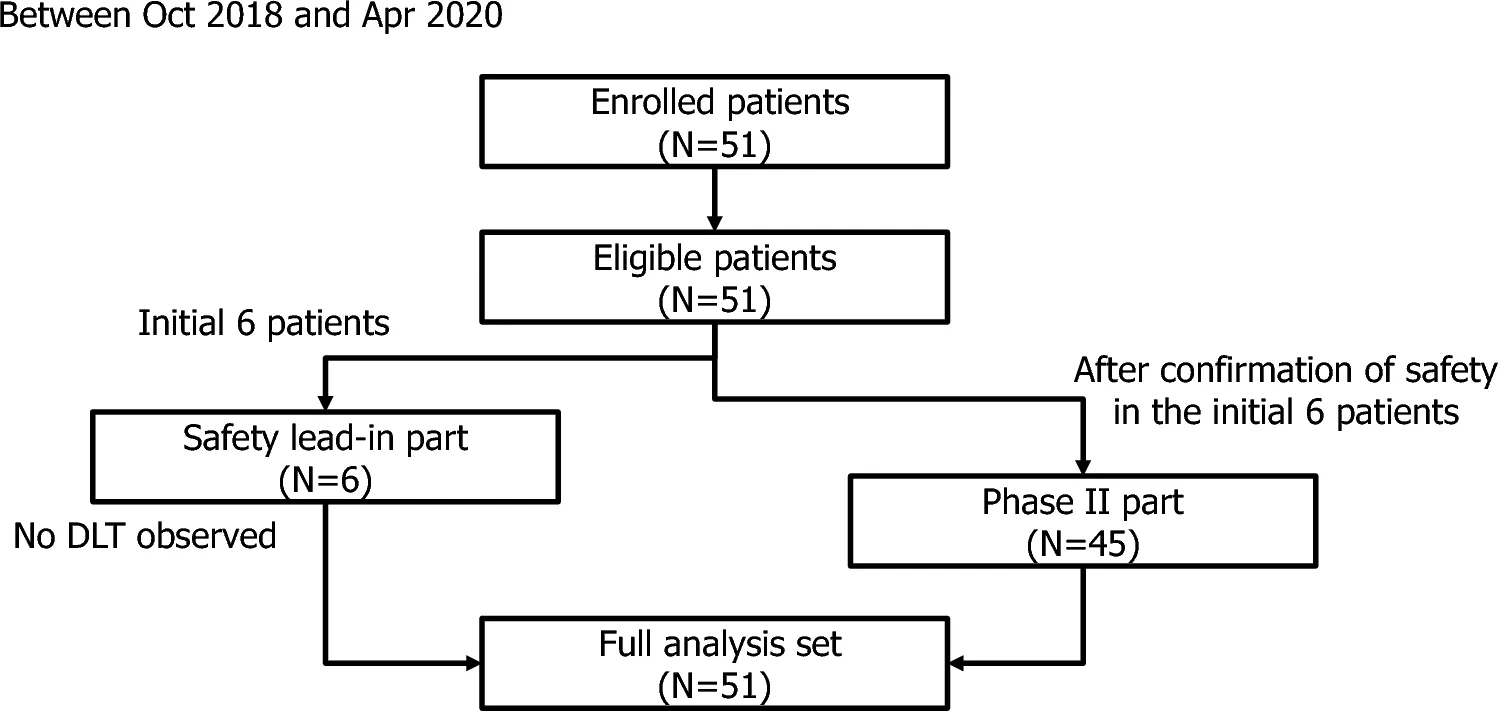
Patient flow diagram. A total of 51 patients were enrolled in the study. The first 6 patients participated in the safety lead-in part, during which no dose-limiting toxicities (DLTs) were observed. Subsequently, 45 additional patients were enrolled. All 51 patients met eligibility criteria and were included in the full analysis set

Patient enrollment began in November 2018. The safety lead-in part was completed by February 2019, with all six patients experiencing no DLTs. Following review by the Efficacy and Safety Evaluation Committee, the study was deemed safe to continue, and full enrollment resumed in April 2019. Enrollment concluded in April 2020, with a 12-month follow-up period after the last patient was enrolled. In total, 51 patients-including those in the safety lead-in part were included. All 51 were designated as the full analysis set, defined as patients who received at least one dose of the investigational drug, met all eligibility criteria, and had no exclusion criteria violations (Fig. 1).

The study cohort characteristics are summarized in Table 1. Briefly, the median age was 65 years (range, 26–74), and 17 patients (33%) were female. A total of 40 patients (78%) had an ECOG PS of 1, while 8 patients (16%) had a PS of 2. Histologically, 38 patients (75%) had the diffuse type. Ten patients (20%) had undergone prior gastrectomy at the time of enrollment. Ascites was present in 50 patients (98%), including 25 (49%) with massive ascites. Inadequate oral intake was observed in 33 patients (65%), including 7 patients (14%) who also had massive ascites.

**Table 1:** Baseline patient characteristics

Characteristics		N = 51 (%)	
Age	Median (years) (range)	65	(26–74)
Sex	Male	34	(67)
	Female	17	(33)
ECOG PS	0	3	(6)
	1	40	(78)
	2	8	(16)
Histological type	Diffuse type	38	(75)
	Intestinal type	12	(24)
	Unknown	1	(2)
HER2 status	Positive	0	(0)
	Negative	38	(75)
	Not evaluated	13	(25)
Disease status	Unresectable	44	(86)
	Recurrent	7	(14)
Prior gastrectomy	Yes	10	(20)
	No	41	(80)
Metastatic sites	Ascites	50	(98)
	Lymph node	16	(31)
	Liver	6	(12)
	Pleura	4	(8)
	Bone	4	(8)
	Ovary	3	(6)
	Lung	1	(2)
Severe peritoneal metastasis	Massive ascites	18	(35)
	Inadequate oral intake	26	(51)
	Both factors	7	(14)

Percentages may not total 100 because of rounding. ECOG, Eastern Cooperative Oncology Group; PS, performance status

### Treatment exposure

The median number of mFOLFOX6 doses administered was 8 (range, 1–26). Median relative dose intensities were as follows: 77.6% for oxaliplatin (33.0 mg/m^2^/week; range, 11.2–43.0), 90.0% for l-leucovorin (90.0 mg/m^2^/week; range, 61.2–103.2), 79.9% for 5-FU bolus (159.8 mg/m^2^/week; range, 77.9–200.8), and 86.5% (for 5-FU continuous infusion 1,038.2 mg/m^2^/week; range, 739.2–1,233.2). At the time of analysis, 49 out of 51 patients had discontinued mFOLFOX6. With a median follow-up duration of 18.1 months (range, 0.2–26.3), the median TTF was 3.8 months (95% CI, 3.0–5.5). Reasons for treatment discontinuation included disease progression (84%), unresolved adverse events beyond 42 days from the last mFOLFOX6 dose (4%), adverse events judged by investigators to warrant discontinuation (4%), patient refusal due to adverse events (2%), patient refusal not related to adverse events (2%), and death unrelated to protocol treatment (4%).

### Efficacy

**Fig. 2:**
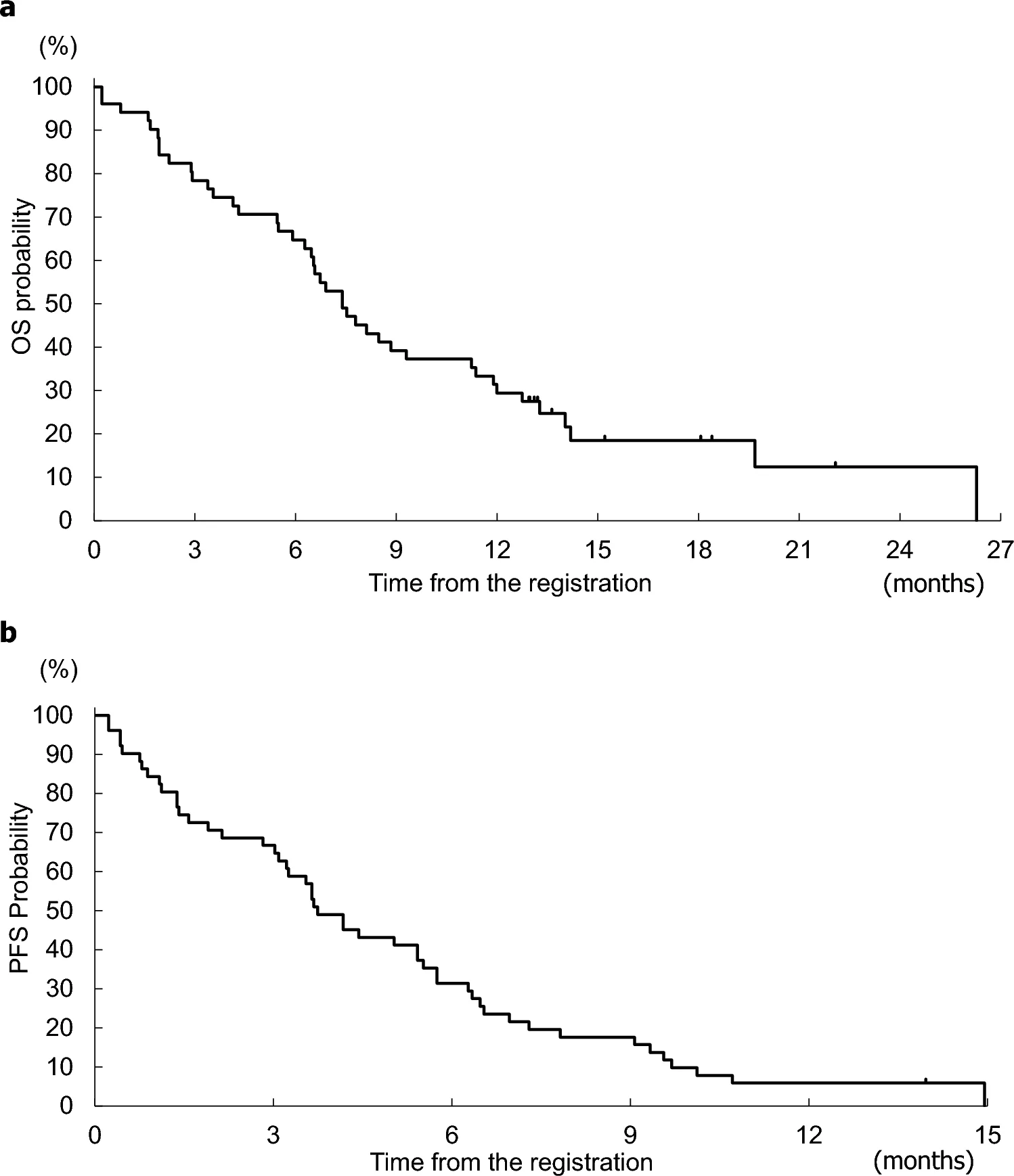
Overall survival and progression-free survival. **A** Median overall survival was 7.4 months (lower limit of one-sided 95% CI 6.5; two-sided 95% CI 5.9–11.2). **B** Median progression-free survival was 3.8 months (95% CI: 3.0–5.5). CI, confidence interval

Among the 51 patients in the full analysis set, the median OS was 7.4 months (lower limit of one-sided 95% CI: 6.5; two-sided 95% CI: 5.9–11.2), indicating that the primary endpoint was met (Fig. 2a). The median PFS was 3.8 months (95% CI: 3.0–5.5) (Fig. 2b). Among 17 patients with measurable lesions, seven achieved a PR, resulting in RR of 41% (95% CI: 18%–67%), while four achieved stable disease, yielding a DCR of 65% (95% CI: 38%–86%). Among 50 patients with ascites, five achieved ascites CR and ten achieved ascites PR, resulting in an ascites RR of 30%. Twenty-one patients had ascites SD, giving an ascites DCR of 72%. The median ascites drainage-free survival was 6.1 months (95% CI: 2.9–7.5). Among 25 patients with massive ascites, nine achieved ascites PR (ascites RR of 36%), and five achieved ascites SD, resulting in an ascites DCR of 56%. During protocol treatment, 11 patients underwent ascites drainage. Nine underwent drainage before the first evaluable post-treatment CT was performed, and their best ascites response was classified as ascites NE. The remaining two patients had already achieved their best ascites response at the first post-treatment CT before subsequent drainage: one achieved ascites PR and underwent the first drainage 197 days after initiation of protocol treatment, and the other achieved ascites SD and underwent the first drainage 284 days after initiation of protocol treatment. Of 33 patients with inadequate oral intake at baseline, 15 (46%) showed improvement. Among those 15 patients, the median time to improvement from mFOLFOX6 initiation was 17 days (range, 4–416), and the median duration of adequate oral intake was 174 days (range, 14–330).

### Adverse events

Among the 51 patients in the safety population, the most common grade 3–4 adverse events were neutropenia (n = 26, 51%), anemia (n = 12, 24%), nausea (n = 8, 16%), anorexia (n = 8, 16%), vomiting (n = 4, 8%), diarrhea (n = 3, 6%), fatigue (n = 3, 6%), and peripheral sensory neuropathy (n = 3, 6%). Febrile neutropenia occurred in one patient (2%) (Table 2). No treatment-related deaths were reported.

**Table 2:** Adverse events

	N = 51			
	All grades (%)		Grade ≥3 (%)	
Neutropenia	37	(73)	26	(51)
Anemia	36	(71)	12	(24)
Thrombocytopenia	26	(51)	1	(2)
Febrile neutropenia	1	(2)	1	(2)
Anorexia	18	(35)	8	(16)
Nausea	32	(63)	8	(16)
Vomiting	19	(37)	4	(8)
Diarrhea	18	(35)	3	(6)
Mucositis	6	(12)	0	(0)
Fatigue	22	(43)	3	(6)
Alopecia	5	(10)	―	―
Peripheral sensory neuropathy	33	(65)	3	(6)
Creatinine increased	8	(16)	0	(0)

### Subsequent treatment

Of the 51 patients, 37 (73%) received second-line treatment. The most common second-line treatments were paclitaxel (PTX) or nab-PTX plus ramucirumab (RAM) (57%), PTX or nab-PTX alone (19%), and anti-PD-1 antibody (11%). The median PFS and OS from the initiation of second-line treatment were 1.8 months and 4.6 months, respectively. Eighteen patients (35%) received third-line treatment. The most common third-line treatments included anti-PD-1 antibody (67%), PTX or nab-PTX plus RAM (17%), and PTX or nab-PTX alone (11%). The median PFS and OS from the start of third-line treatment were 1.1 months and 2.7 months, respectively.

### Comparison of survival and safety with the JCOG1108/WJOG7312G cohort treated with FLTAX or FL

Of the 51 patients treated with FL and 48 patients treated with FLTAX in the JCOG1108/WJOG7312G study, we analyzed survival in 48 and 47 patients, respectively, after excluding those with ECOG PS of 2 and both massive ascites and inadequate oral intake who were not eligible for the current WJOG10517G study. Baseline patient characteristics of the FL, FLTAX, and mFOLFOX6 groups included in the individual patient data comparative analysis are shown in Supplementary Table 1 (Online Resource 2). The propensity score-weighted analysis included 47 patients in the FL group, 47 in the FLTAX group, and 48 in the mFOLFOX6 group after excluding patients with missing values for any propensity score covariate.

**Fig. 3:**
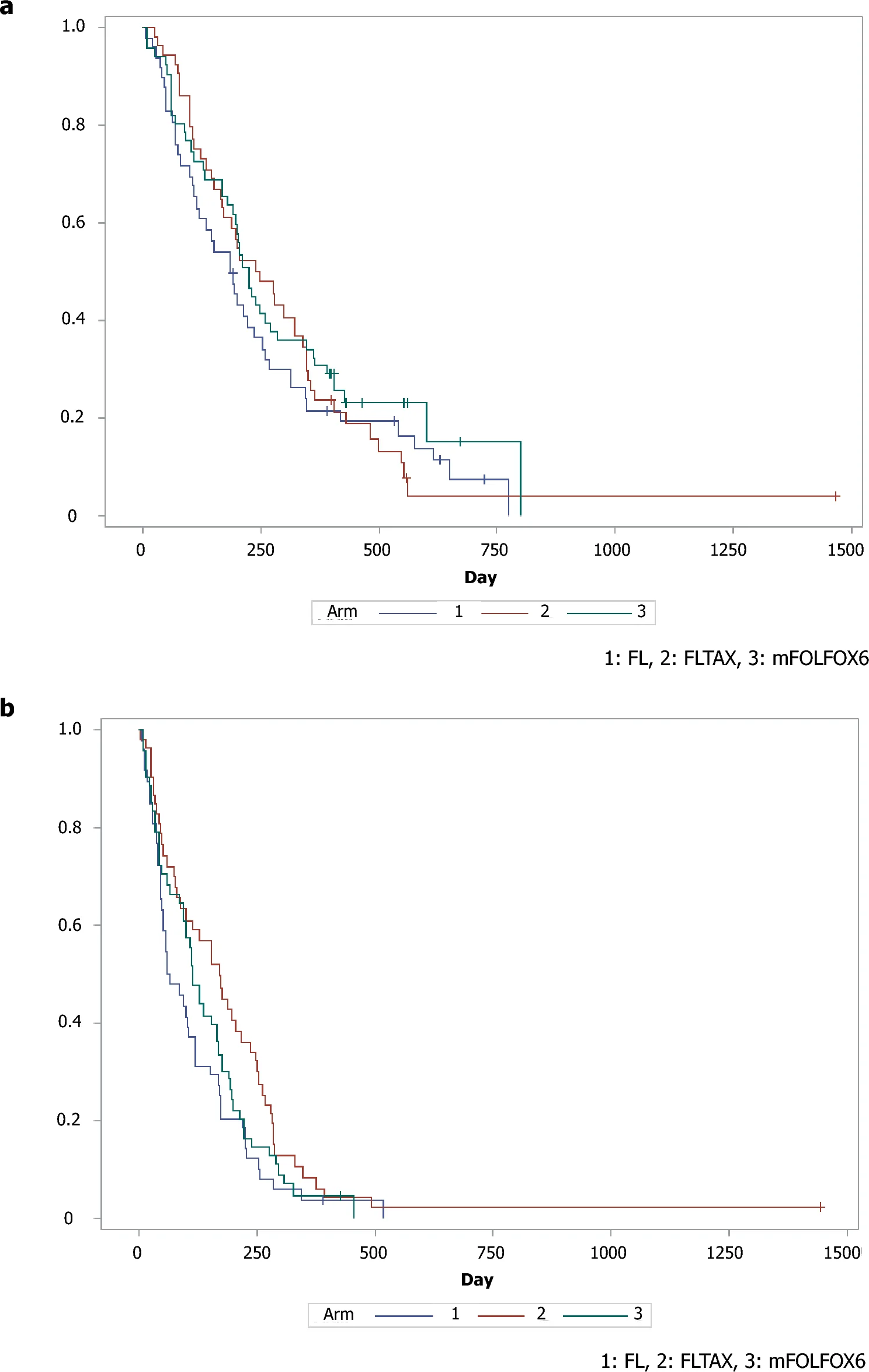
Comparative survival analysis of individual patient data: mFOLFOX6 vs. FL or FLTAX (JCOG1108/WJOG7312G cohort). **A** Overall survival with mFOLFOX6 tended to be longer than with FL (HR, weighted by propensity score: 0.76; 95% CI 0.56–1.03; *p* = 0.08) and was comparable to that with FLTAX (HR: 0.87; 95% CI 0.64–1.18; *p* = 0.38). **B** Progression-free survival with mFOLFOX6 was similar to FL (HR: 0.83; 95% CI 0.62–1.10; *p* = 0.20), and tended to be shorter than with FLTAX (HR: 1.33; 95% CI 0.99–1.78; *p* = 0.06). *CI* confidence interval; *HR* hazard ratio

OS for mFOLFOX6 was comparable to that of FL, with a crude hazard ratio (HR) of 0.85 (95% CI: 0.55–1.31; *p* = 0.47), and showed a trend toward improvement when adjusted using propensity score weighting, with an HR of 0.76 (95% CI: 0.56–1.03; *p* = 0.08). OS for mFOLFOX6 was also similar to that of FLTAX, with both the crude HR (0.89, 95% CI: 0.58–1.36; *p* = 0.59) (Supplementary Fig. 1a; Online Resource 1) and the propensity score–weighted HR (0.87; 95% CI: 0.64–1.18; *p* = 0.38) (Fig. 3a) showing no significant differences. PFS for mFOLFOX6 was also comparable to that of FL, with a crude HR of 0.79 (95% CI: 0.53–1.18; *p* = 0.24) and a propensity score–weighted HR of 0.83 (95% CI: 0.62–1.10; *p* = 0.20). Similarly, PFS for mFOLFOX6 was comparable to that for FLTAX based on the crude HR of 1.22 (95% CI: 0.82–1.84; *p* = 0.32) (Supplementary Fig. 1b; Online Resource 1), though it tended to be shorter when weighted by the propensity score, with an HR of 1.33 (95% CI: 0.99–1.78; *p* = 0.06) (Fig. 3b).

The comparative safety analysis included 48, 46, and 51 patients in the FL, FLTAX, and mFOLFOX6 groups, respectively. One of the 47 patients in the FLTAX efficacy cohort did not initiate protocol treatment and was therefore excluded from the safety analysis. Adverse events in the three groups are summarized in Supplementary Table 2 (Online Resource 3). Grade 3 or higher neutropenia occurred in 13 patients (27%), 9 patients (20%), and 26 patients (51%) in the FL, FLTAX, and mFOLFOX6 groups, respectively, while febrile neutropenia occurred in 2 patients (4%), 2 patients (4%), and 1 patient (2%), respectively.

## Discussion

In the present phase II study, we demonstrated that mFOLFOX6 may be one of the treatment options for AGC patients with SPM. To our knowledge, this is the first prospective study to show the efficacy of mFOLFOX6 in AGC patients with SPM.

The JCOG1108/WJOG7312G study showed that FLTAX may serve as a first-line treatment option for AGC patients with SPM [8]. The Japanese Gastric Cancer Treatment Guidelines weakly recommend chemotherapy for patients with impaired oral intake or massive ascites due to extensive peritoneal disease after careful assessment of their general condition [12]. However, first-line treatment for AGC patients without SPM is now being developed with fluoropyrimidine plus platinum, including oxaliplatin, as the backbone regimen. Since the individual patient data comparative analysis from this study suggested that the OS of mFOLFOX6 may be comparable to that of FLTAX, the present results support the development of mFOLFOX6 as a backbone regimen not only for AGC patients without but also for those with SPM, aiming to improve outcomes in this population. Although PFS with mFOLFOX6 tended to be shorter than with FLTAX, a higher proportion of patients receiving mFOLFOX6 (73%) went on to second-line treatment, mainly PTX-based compared to 57% of those treated with FLTAX, who mainly received oxaliplatin- or irinotecan-based regimens. This likely contributed to the comparable OS between the two groups. Nivolumab plus mFOLFOX6 is the standard first-line treatment for AGC patients without SPM [1], while zolbetuximab plus mFOLFOX6 is standard for CLDN18.2 positive AGC patients without SPM [5]. The efficacy of adding nivolumab or zolbetuximab to mFOLFOX6 should also be evaluated in AGC patients with SPM. Based on the findings of the current study, we initiated a phase II trial of nivolumab plus mFOLFOX6 in this patient group (WJOG16322G, jRCTs041220164), which is currently ongoing [13]. An individual patient data comparative analysis of the present WJOG10517G and WJOG16322G studies is also being planned.

A phase II study of intraperitoneal paclitaxel plus mFOLFOX6 has previously been reported in AGC patients with peritoneal metastases and inadequate oral intake [14]. Inadequate oral intake was one of the two factors defining SPM in the present study. In that study, the median PFS and OS were 7.7 and 12.4 months, respectively. However, the study did not clearly define “inadequate oral intake” and excluded patients with massive ascites, suggesting that its study population may have had a more favorable prognosis than that of the present study. Therefore, although the favorable outcomes observed in that study are encouraging, they cannot be directly extrapolated to AGC patients with SPM as defined in WJOG10517G. Further investigation of intraperitoneal paclitaxel plus mFOLFOX6 in patients meeting eligibility criteria similar to those of WJOG10517G is warranted.

The frequency of grade 3 or higher neutropenia associated with mFOLFOX6 was greater in the present study compared with previous reports involving AGC patients without SPM [1]. This may be due to differences in the general condition of enrolled patients: only 6% of patients in the present study had an ECOG PS of 0, compared with approximately 50% in earlier reports. However, other adverse events, including febrile neutropenia, associated with mFOLFOX6 were comparable between the present study and previous data. In an individual patient data safety analysis restricted to patients meeting the WJOG10517G eligibility criteria who initiated protocol treatment, grade 3 or higher neutropenia occurred in 51% of patients receiving mFOLFOX6, compared with 27% receiving FL and 20% receiving FLTAX. In contrast, febrile neutropenia occurred in 2%, 4%, and 4% of patients, respectively. Thus, although severe neutropenia was more frequent with mFOLFOX6, febrile neutropenia was not more frequent than with FL or FLTAX. These findings support further development of mFOLFOX6 as a backbone regimen for AGC patients with SPM.

The present study has several limitations. First, it was a single-arm phase II trial; therefore, definitive conclusions cannot be drawn from its findings. To address the inherent limitations of this design, we conducted a prespecified individual patient data comparative analysis using data from the JCOG1108/WJOG7312G study, which employed identical eligibility and exclusion criteria for FL and FLTAX. Second, differences in the distribution of prognostic factors may have influenced outcomes and could not be fully matched to those in the historical control group. To minimize this bias, propensity score weighting using IPTW was applied during the individual patient data analysis to adjust for differences in baseline characteristics between the two studies.

## Conclusions

This phase II study demonstrated that mFOLFOX6 was tolerable and may serve as one of the treatment options for AGC patients with SPM. These findings support the further development of mFOLFOX6 as a backbone regimen not only for AGC patients with SPM but also for those without, with the goal of improving prognosis in this patient population.

## Supplementary Information

Below is the link to the electronic supplementary material.

Supplementary file1 (PDF 342 KB)

Supplementary file2 (PDF 144 KB)

Supplementary file3 (PDF 140 KB)

## Data Availability

The datasets generated and/or analyzed during this study are not publicly available but are available from the corresponding author upon reasonable request.
