# Supplementary material for: Phase II study of mFOLFOX6 in advanced gastric cancer patients with severe peritoneal metastases (WJOG10517G), including an individual patient data comparison with JCOG1108/WJOG7312G
Source: Gastric Cancer. 2026 Aug 27;29(6):1327–36. doi: 10.1007/s10120-026-01790-3 (PMC13755017; doi:10.1007/s10120-026-01790-3)
Supplement: Supplementary file 1 [file 10120_2026_1790_MOESM1_ESM.pdf]

Electronic Supplementary Material

Supplementary Figure 1

Article title

Phase II study of mFOLFOX6 in advanced gastric cancer patients with severe peritoneal metastases (WJOG10517G), including an individual patient data comparison with JCOG1108/WJOG7312G

Journal

Gastric Cancer

Authors

Toshiki Masuishi, Takayuki Ando, Hiroki Hara, Takeshi Kawakami, Yoshiyuki Yamamoto, Naotoshi Sugimoto, Kazuhiro Shiraishi, Taito Esaki, Yuji Negoro, Takao Tsuzuki, Hiroaki Sawai, Masato Nakamura, Takashi Inagaki, Yudai Shinohara, Kentaro Kawakami, Seiichiro Mitani, Kenji Tsuchihashi, Chihiro Kondoh, Hiroo Katsuya, Osamu Maeda, Yuriko Fujita, Kenichi Yoshimura, Masanori Terashima, Takako Nakajima Eguchi, and Kei Muro

Corresponding author

Toshiki Masuishi

Affiliation

Department of Clinical Oncology, Aichi Cancer Center Hospital, Nagoya, Japan

E-mail

tmasuishi@aichi-cc.jp

Supplementary Figure

Fig. S1a. Comparison of overall survival with the JCOG1108/WJOG7312G cohort treated with FLTAX or FL.

Overall survival for mFOLFOX6 was comparable to that of FL with a crude hazard ratio (HR) of 0.85 and was also similar to that of FLTAX, with the crude HR of 0.89.

Fig. S1b. Comparison of progression-free survival with the JCOG1108/WJOG7312G cohort treated with FLTAX or FL.

Progression-free survival for mFOLFOX6 was also comparable to that of FL with a crude HR of 0.79 and was comparable to that for FLTAX based on the crude HR of 1.22.

Fig. S1a

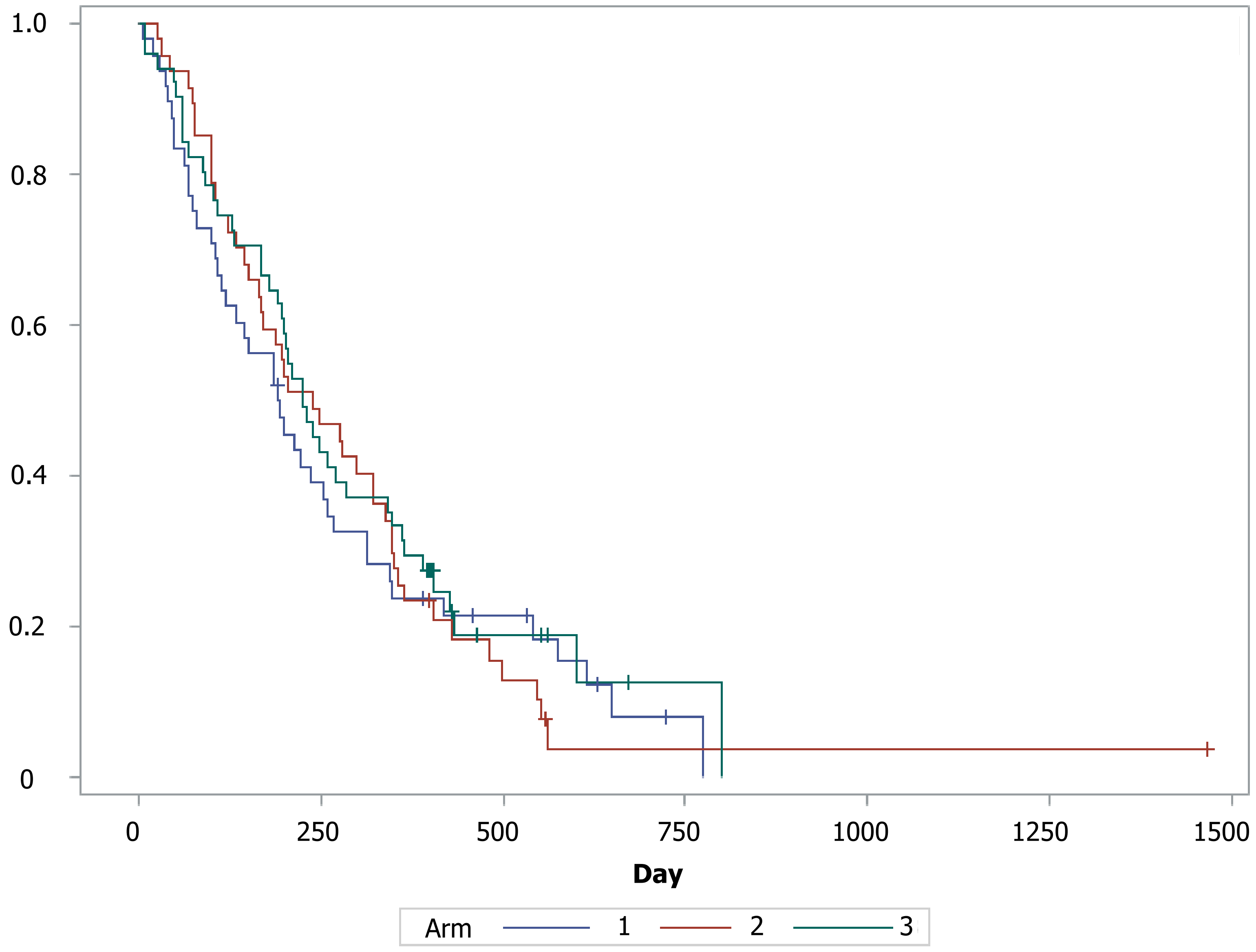

1: FL, 2: FLTAX, 3: mFOLFOX6

Fig. S1b

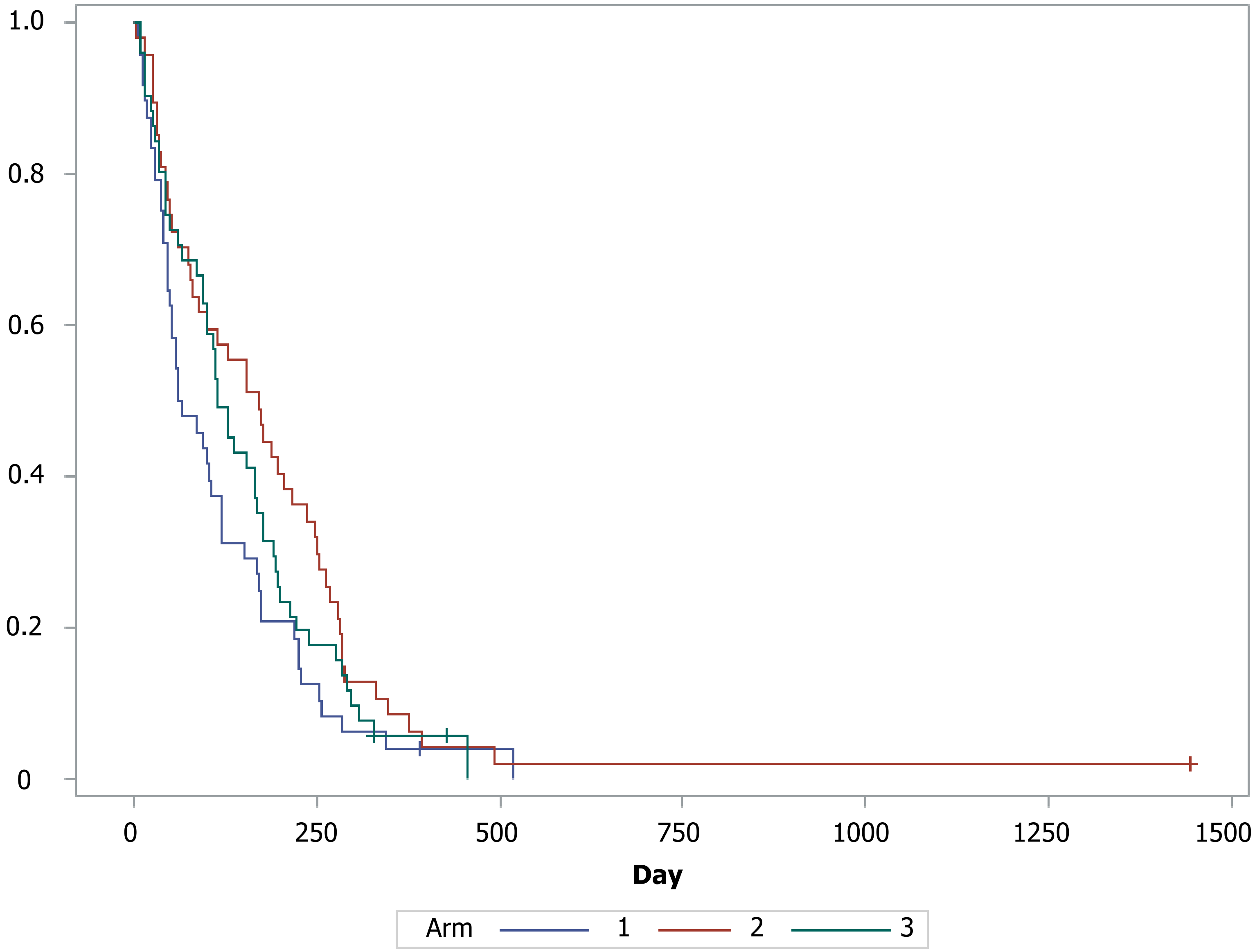

1: FL, 2: FLTAX, 3: mFOLFOX6
