# Supplementary material for: Phase II study of mFOLFOX6 in advanced gastric cancer patients with severe peritoneal metastases (WJOG10517G), including an individual patient data comparison with JCOG1108/WJOG7312G
Source: Gastric Cancer. 2026 Aug 27;29(6):1327–36. doi: 10.1007/s10120-026-01790-3 (PMC13755017; doi:10.1007/s10120-026-01790-3)
Supplement: Supplementary file 2 [file 10120_2026_1790_MOESM2_ESM.pdf]

Electronic Supplementary Material

Supplementary Table 1

Article title

Phase II study of mFOLFOX6 in advanced gastric cancer patients with severe peritoneal metastases (WJOG10517G), including an individual patient data comparison with JCOG1108/WJOG7312G

Journal

Gastric Cancer

Authors

Toshiki Masuishi, Takayuki Ando, Hiroki Hara, Takeshi Kawakami, Yoshiyuki Yamamoto, Naotoshi Sugimoto, Kazuhiro Shiraishi, Taito Esaki, Yuji Negoro, Takao Tsuzuki, Hiroaki Sawai, Masato Nakamura, Takashi Inagaki, Yudai Shinohara, Kentaro Kawakami, Seiichiro Mitani, Kenji Tsuchihashi, Chihiro Kondoh, Hiroo Katsuya, Osamu Maeda, Yuriko Fujita, Kenichi Yoshimura, Masanori Terashima, Takako Nakajima Eguchi, and Kei Muro

Corresponding author

Toshiki Masuishi

Affiliation

Department of Clinical Oncology, Aichi Cancer Center Hospital, Nagoya, Japan

E-mail

tmasuishi@aichi-cc.jp

Supplementary Table 1. Baseline patient characteristics in the individual patient data comparative analysis

Characteristics, n (%)		FL (N = 48)	FLTAX (N = 47)	mFOLFOX6 (N = 51)
Age	Median (range), years	64 (41–75)	65 (29–75)	65 (26–74)
Sex	Male	32 (67)	28 (60)	34 (67)
	Female	16 (33)	19 (40)	17 (33)
ECOG PS	0–1	38 (79)	36 (77)	43 (84)
	2	10 (21)	11 (23)	8 (16)
Factors contributing to SPM	Massive ascites only	26 (54)	23 (49)	18 (35)
	Inadequate oral intake only	15 (31)	18 (38)	26 (51)
	Both factors	7 (15)	6 (13)	7 (14)
Number of SPM factors	1	41 (85)	41 (87)	44 (86)
	2	7 (15)	6 (13)	7 (14)
Disease status	Unresectable	39 (81)	40 (85)	44 (86)
	Recurrent	9 (19)	7 (15)	7 (14)
Histological type	Intestinal type	14 (29)	8 (17)	12 (24)
	Diffuse type	34 (71)	39 (83)	38 (75)
	Missing	0 (0)	0 (0)	1 (2)
WBC, /μL	<8,600	34 (71)	33 (70)	37 (73)
	≥8,600	14 (29)	14 (30)	14 (27)
ALP, U/L	<322	36 (75)	37 (79)	40 (78)
	≥322	12 (25)	10 (21)	9 (18)
	Missing	0 (0)	0 (0)	2 (4)
mGPS	0–1	25 (52)	25 (53)	30 (59)
	2	22 (46)	22 (47)	21 (41)
	Missing	1 (2)	0 (0)	0 (0)

Percentages may not total 100 because of rounding. ECOG, Eastern Cooperative Oncology Group; PS, performance status; SPM, severe peritoneal metastases; WBC, white blood cell count; ALP, alkaline phosphatase; mGPS, modified Glasgow Prognostic Score; FL, 5-fluorouracil/l-leucovorin; FLTAX, FL plus paclitaxel.
