# Supplementary material for: Phase II study of mFOLFOX6 in advanced gastric cancer patients with severe peritoneal metastases (WJOG10517G), including an individual patient data comparison with JCOG1108/WJOG7312G
Source: Gastric Cancer. 2026 Aug 27;29(6):1327–36. doi: 10.1007/s10120-026-01790-3 (PMC13755017; doi:10.1007/s10120-026-01790-3)
Supplement: Supplementary file 3 [file 10120_2026_1790_MOESM3_ESM.pdf]

Electronic Supplementary Material

Supplementary Table 2

Article title

Phase II study of mFOLFOX6 in advanced gastric cancer patients with severe peritoneal metastases (WJOG10517G), including an individual patient data comparison with JCOG1108/WJOG7312G

Journal

Gastric Cancer

Authors

Toshiki Masuishi, Takayuki Ando, Hiroki Hara, Takeshi Kawakami, Yoshiyuki Yamamoto, Naotoshi Sugimoto, Kazuhiro Shiraishi, Taito Esaki, Yuji Negoro, Takao Tsuzuki, Hiroaki Sawai, Masato Nakamura, Takashi Inagaki, Yudai Shinohara, Kentaro Kawakami, Seiichiro Mitani, Kenji Tsuchihashi, Chihiro Kondoh, Hiroo Katsuya, Osamu Maeda, Yuriko Fujita, Kenichi Yoshimura, Masanori Terashima, Takako Nakajima Eguchi, and Kei Muro

Corresponding author

Toshiki Masuishi

Affiliation

Department of Clinical Oncology, Aichi Cancer Center Hospital, Nagoya, Japan

E-mail

tmasuishi@aichi-cc.jp

Supplementary Table 2. Adverse events in the individual patient data comparative analysis

Adverse event, n (%)	FL (N = 48)		FLTAX (N = 46)		mFOLFOX6 (N = 51)	
	All grades (%)	Grade ≥3 (%)	All grades (%)	Grade ≥3 (%)	All grades (%)	Grade ≥3 (%)
Neutropenia	28 (58)	13 (27)	27 (59)	9 (20)	37 (73)	26 (51)
Anemia	47 (98)	6 (13)	45 (98)	12 (26)	36 (71)	12 (24)
Thrombocytopenia	18 (38)	0 (0)	7 (15)	1 (2)	26 (51)	1 (2)
Febrile neutropenia	2 (4)	2 (4)	2 (4)	2 (4)	1 (2)	1 (2)
Anorexia	42 (88)	22 (46)	36 (78)	14 (30)	18 (35)	8 (16)
Nausea	31 (65)	4 (8)	22 (48)	4 (9)	32 (63)	8 (16)
Vomiting	17 (35)	3 (6)	19 (41)	2 (4)	19 (37)	4 (8)
Diarrhea	24 (50)	3 (6)	21 (46)	3 (7)	18 (35)	3 (6)
Mucositis	9 (19)	0 (0)	5 (11)	0 (0)	6 (12)	0 (0)
Fatigue	38 (79)	8 (17)	33 (72)	5 (11)	22 (43)	3 (6)
Alopecia	2 (4)	—	25 (54)	—	5 (10)	—
Peripheral sensory neuropathy	1 (2)	0 (0)	17 (37)	3 (7)	33 (65)	3 (6)
Creatinine increased	22 (46)	2 (4)	21 (46)	2 (4)	8 (16)	0 (0)

AE, adverse event; FL, 5-fluorouracil/l-leucovorin; FLTAX, FL plus paclitaxel.
